# Non-metastatic pediatric head and neck rhabdomyosarcoma: a 24-year retrospective experience from Jordan

**DOI:** 10.3389/fped.2026.1899565

**Published:** 2026-07-16

**Authors:** Arwa Kiswani, Omar Jaber, Ahmad Kh. Ibrahimi, Nasim Sarhan, Yacoub A. Yousef, Mona Mohammad, Rula Al-Qawabah, Iyad Sultan, Hadeel Halalsheh

**Affiliations:** 1 Department of Pediatrics, King Hussein Cancer Center, Amman, Jordan; 2 Department of Pathology, King Hussein Cancer Center, Amman, Jordan; 3 Department of Radiation Oncology, King Hussein Cancer Center, Amman, Jordan; 4 Department of Surgery, Ophthalmology, King Hussein Cancer Center, Amman, Jordan; 5 Department of Radiology, King Hussein Cancer Center, Amman, Jordan; 6 Artificial Intelligence Office, King Hussein Cancer Center, Amman, Jordan; 7 Department of Pediatrics, The University of Jordan, Amman, Jordan

**Keywords:** local control, MENA, parameningeal, pediatric oncology, resource-limited settings, rhabdomyosarcoma, survival

## Abstract

**Background:**

Pediatric head and neck rhabdomyosarcoma (HNRMS) is well characterized in Western cooperative group trials, but real-world outcomes from resource-constrained MENA settings are poorly documented. Whether the locally advanced-stage presentation typical of Middle Eastern referral centers precludes outcomes equivalent to international benchmarks is unknown.

**Methods:**

We conducted a retrospective analysis of pediatric patients younger than 18 years with non-metastatic HNRMS diagnosed between January 2001 and May 2025. We extracted demographic, pathological, treatment, and outcome data. Risk stratification and treatment planning followed COG protocols throughout, with local control decisions made via multidisciplinary team assessment.

**Results:**

Among 98 patients with HNRMS, 77 (79%) had non-metastatic disease and were included in the analysis. The male-to-female ratio was 1:1, with a median age at diagnosis of 5.9 years (range, 0.2–18). The parameningeal region (*n* = 31, 40%) and the orbital region (*n* = 20, 26%) were the most common primary sites. Histology was embryonal in 51 patients (66%), and tumor size exceeded 5 cm in 55 patients (71%). Sixty patients (78%) were classified as intermediate risk and 93.5% were IRS Clinical Group III. All patients received chemotherapy, with 32% receiving maintenance therapy. Local excision was performed in 15 patients (19%), and 71 patients (92%) received radiotherapy. Twenty-three patients (30%) experienced relapse or progression, with 74% presenting with local recurrence or progression. The median follow-up time was 30.7 months (range, 2.6–294). The 5-year event-free survival (EFS) and overall survival (OS) were 66.1% (55.4%–79%) and 70.1% (59.1–83.1%), respectively. Subgroup analysis demonstrated variable 5-year OS rates according to primary site: orbital (91.7 ± 8%), parameningeal (51.2 ± 10.6**%**), and other HN primaries (76.1 ± 9.4**%**).

**Conclusion:**

Despite 93.5% IRS Clinical Group III and 71% tumors >5 cm—rates substantially exceeding Western cooperative group series—5-year OS of 70.1% was achieved. Parameningeal location was the only factor significantly associated with adverse survival outcomes in this cohort, driven by local failure. These findings demonstrate that outcomes equivalent to international benchmarks are attainable in a resource-constrained MENA setting, and identify local control intensification for parameningeal disease as the priority unmet need.

## Introduction

Rhabdomyosarcoma (RMS) is the most common soft-tissue sarcoma in children and adolescents (1). It accounts for nearly half of all pediatric soft-tissue sarcomas, and due to its biological heterogeneity and variable site predilection, remains challenging to manage consistently.

The anatomical distribution of RMS varies considerably with age. In children, RMS most frequently arises in the head and neck region, followed by the genitourinary tract, and the extremities (1, 2). Conversely, RMS in adults is relatively uncommon and is more often found in extremities, with rare occurrences in the head and neck area (3). This anatomical distribution is not solely determined by age; it is also strongly influenced by histologic and molecular subtype. Embryonal and alveolar RMS differ in both age distribution and site predilection and are biologically distinct entities despite sharing the RMS designation. The observed site-related patterns therefore likely reflect, at least in part, the underlying subtype composition of the population. This variability highlights the importance of age-specific and site-specific characterization of the disease to improve diagnosis, risk stratification, and therapeutic planning.

Whereas HNRMS is well characterized by North American and European cooperative groups, real-world data from the Middle East and North Africa (MENA) region, where referral patterns, diagnostic delays, and resource availability may affect presentation stage and adherence to protocol, are scarce.

King Hussein Cancer Center (KHCC) is the primary national referral center for pediatric oncology in Jordan, treating patients from across the country and neighboring regions. We hypothesized that the disproportionate advanced local stage presentation documented in this cohort would translate to inferior outcomes compared with cooperative group benchmarks, and aimed to identify which clinical factors –primary site, tumor burden, and local control strategy– modulate outcomes in this resource-constrained setting.

## Materials and methods

With KHCC institutional review board's approval (IRB 24-KHCC-044), a retrospective analysis of medical records for pediatric patients aged 18 or younger, who were newly diagnosed with RMS, was performed. This study focused on patients treated at our center from January 2001 to May 2025; patients were followed until January 2026. Patient data were extracted from the KHCC institutional electronic medical records system and the KHCC Cancer Registry, which prospectively captures all cancer diagnoses managed at the center. Data extracted included demographic information, pathology reports, imaging studies, treatment records, and follow-up documentation. Anatomical sites for HNRMS were categorized into three subgroups: orbital, parameningeal (deep head and neck sites adjacent to the meninges, including the nasopharynx, paranasal sinuses, middle ear, and infratemporal fossa), and other non-parameningeal/non-orbital sites (such as the scalp, parotid, or oral cavity). Tumor size was categorized as either greater or less than 5 cm at the time of initial diagnosis.

Pathological diagnoses were established by KHCC institutional pathologists according to the World Health Organization (WHO) classification criteria applicable at the time of diagnosis. Historical diagnoses were not systematically re-reviewed according to contemporary WHO criteria. Initially, diagnoses were based on histopathology and immunohistochemistry alone. Starting in 2018, FOXO1 fusion testing by fluorescence *in situ* hybridization (FISH) was done initially for alveolar subtypes and eventually for all patients. Cases classified as RMS not otherwise specified (NOS) represent tumors in which morphological and immunohistochemical features were consistent with RMS but insufficient for definitive subtype assignment under classification criteria; central pathology review was not performed for this retrospective cohort.

Since metastasis is the strongest prognostic factor in RMS, the study included only patients with non-metastatic HNRMS, and excluding those who only sought consultation or underwent surgery/radiotherapy without additional treatment.

Tumor location and local extent at diagnosis were evaluated using computed tomography (CT) or magnetic resonance imaging (MRI), depending on the disease site. Initial staging investigations included a CT scan of the chest, bone scan, and bilateral bone marrow biopsies (or PET scan, if available).

The risk stratification and treatment plan followed the published Children's Oncology Group (COG) study protocols at the time of treatment, with patients categorized by their risk level into low-risk (LR), intermediate-risk (IR), and high-risk (HR) groups. Primary surgical resection was pursued when gross tumor removal could be achieved without significant morbidity; in cases where this was not feasible, a biopsy was performed to establish the diagnosis, followed by neo-adjuvant chemotherapy.

Local control was achieved using surgery, radiotherapy, or a combination of both, depending on the resectability of tumor. Strategies were determined via multidisciplinary team (MDT) assessment based on tumor site, resectability, predicted morbidity, and chemotherapy response, aligning with COG and EpSSG guidelines. Definitive radiotherapy (RT) was favored for orbital tumors to avoid enucleation and disfigurement, and served as the cornerstone for parameningeal tumors due to skull base and neurovascular proximity. For other sites, surgery (delayed primary excision) was pursued only if gross total resection with acceptable morbidity was achievable; otherwise, RT was utilized for unresectable or incompletely resected disease.

RT for local control, when indicated, was given at week 12. Dosing followed COG guideline recommendations using the pre-chemotherapy tumor volume plus a standard anatomical margin for the Clinical Target Volume. It was delivered using conformal techniques, predominantly utilizing 3D conformal radiation therapy (3D-CRT) or intensity-modulated radiation therapy (IMRT). Chemotherapy regimens adhered to COG protocols, most commonly incorporating Vincristine, Actinomycin-D, and Cyclophosphamide (VAC). Patients received a cyclophosphamide dose of 2.2 g/m^2^ until May 2006, after which the protocol was updated to a reduced dose of 1.2 g/m^2^ for all patients, based on our previously published institutional experience demonstrating reduced toxicity without compromising survival outcomes (4). This dosing schedule was applied uniformly across all primary sites. From 2018 onwards, all patients with IR and HR rhabdomyosarcoma received six months of maintenance therapy with vinorelbine and oral cyclophosphamide as per EpSSG guidelines.

### Statistical analysis

Demographic, tumor, and treatment characteristics were summarized by descriptive analysis. Categorical variables were compared using Pearson's Chi-squared or Fisher's exact tests, while continuous variables were analyzed using the Wilcoxon rank-sum test. Overall survival (OS) was defined as the time between diagnosis and death from any cause or last follow-up for patients remaining alive. Event-free survival (EFS) was defined as the time between diagnosis and occurrence of disease recurrence or progression, second malignancy, death, or last follow-up for patients who did not experience an event. OS and EFS were calculated using the Kaplan–Meier method. The log-rank test was used to compare survival curves when needed. Cox proportional hazards regression was performed for univariable and multivariable analyses to identify factors associated with EFS and OS. Hazard ratios (HRs) with 95% confidence intervals (CIs) and *p*-values were reported. A *p* value of 0.05 or less was considered statistically significant.

## Results

Between January 2001 and May 2025, a total of 224 patients were diagnosed with RMS. Of these, 98 (43.8%) presented with head and neck tumors (HNRMS), and 77 of those without metastatic disease met the inclusion criteria for this analysis.

The median age at diagnosis was 5.9 years (IQR: 4.0–9.8; range, 0.2–18). Most patients were diagnosed between 1 and 9 years of age (70%), while 5.2% were younger than 1 year. The cohort showed a balanced sex distribution, with 39 (51%) female patients; tumor characteristics are summarized in Table 1.

**Table 1:** Characteristics of patients and tumors.

Variable	All patients, number (percentage)
Total patients	77
Age at diagnosis median (range) years	5.9 (0.2–18)
Age category	
<1 year	4 (5.2%)
1–9 years	54 (70%)
≥10 years	19 (25%)
Sex	
Females	39 (51%)
Males	38 (49%)
Primary site	
Parameningeal	31 (40%)
Orbital	20 (26%)
Non-PM Non-Orbital	26 (34%)
Histological subtype	
Alveolar	18 (23.4%)
Embryonal	51 (66.2%)
Rhabdomyosarcoma, NOS^a^	8 (10.4%)
FOXO1 test	
Positive	3 (3.9%)
Negative	22 (28.6%)
Not done	52 (67.5%)
Tumor size	
<5 cm	22 (29%)
≥5 cm	55 (71%)
Clinical group	
I	2 (2.6%)
II	3 (3.9%)
III	72 (93.5%)
Stage	
1	46 (59.7%)
2	7 (9.1%)
3	24 (31.2%)
Regional lymph node by scans	12 (16%)
Risk group	
Intermediate risk	60 (78%)
Low risk	17 (22%)
Treatment	
Surgery	15 (19%)
Radiation therapy	71 (92%)
Chemotherapy	77 (100%)
VAC only	63 (82%)
VAC/VI	14 (18%)

a NOS includes 2 spindle cell RMS cases.

PM, parameningeal; VAC; vincristine, actinomycin, cyclophosphamide, VI; vincristine, irinotecan.

Parameningeal tumors were the most common primary site (*n* = 31, 40%), followed by non-parameningeal/non-orbital sites (*n* = 26, 34%) and orbital tumors (*n* = 20, 26%). Embryonal rhabdomyosarcoma was the predominant histologic subtype (*n* = 51, 66%), followed by alveolar RMS (*n* = 18, 23%), RMS NOS (*n* = 6, 7.8%), and spindle cell RMS (*n* = 2, 2.6%). Tumor size ≥ 5 cm was present in 55 patients (71%), and regional nodal involvement on imaging was documented in 12 patients (16%).

Clinical risk stratification according to COG criteria classified 60 patients (78%) as intermediate-risk. According to pretreatment TNM staging, 46 patients (59.7%) were classified as Stage 1, 7 patients (9.1%) as Stage 2, and 24 patients (31.2%) as Stage 3. Based on the extent of initial surgical resection, most patients (*n* = 72, 93.5%) fell into Intergroup Rhabdomyosarcoma Study (IRS) Clinical Group III (Table 1). FOXO1 fusion testing was performed in 25 patients, and was negative in 22 (28.6% of the whole cohort). Among the five patients younger than one year at diagnosis (infantile cases), FOXO1 fusion testing was performed in two, both of whom were fusion-negative. Given the small number of infantile cases and the unavailability of comprehensive genomic profiling in our retrospective cohort, a full molecular characterization of this subgroup was not possible.

All patients received systemic chemotherapy. The VAC only regimen was most frequently administered (82%), followed by VAC/VI (vincristine, irinotecan) (18%). Maintenance therapy was given in 32% of cases. Regarding the cyclophosphamide dose, nine patients (12%) received the 2.2 g/m^2^ dose, while 68 patients (88%) received the 1.2 g/m^2^ dose.

Surgical resection was performed in 15 patients (19%). Of these, negative margins were achieved in 8 (53.3%) and positive margins in 7 (46.7%). Radiotherapy was delivered to 71 patients (92%) and the median dose was 50.4 Gy (range, 30–50.4).

**Figure 1:**
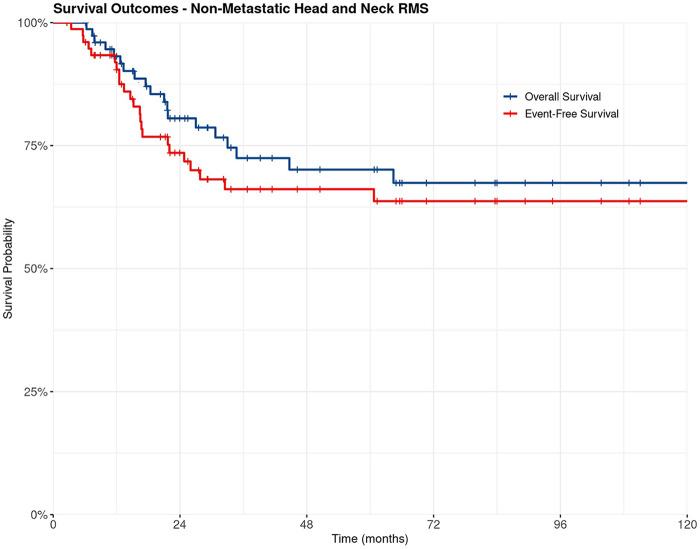
Kaplan–Meier 5-year event free survival and overall survival for all patients with non-metastatic head and neck rhabdomyosarcoma.

At a median follow-up of 30.7 months (range, 2.6–294), 23 patients (29.9%) had experienced an event, and 19 (24.7%) had died. The estimated 5-year EFS was 66.1% (95% CI: 55.4–79.0), and the 5-year OS was 70.1% (95% CI: 59.1–83.1) (Figure 1). Local recurrence was the predominant pattern of treatment failure, affecting 16 patients (20.8%). Distant failures were less common, including isolated relapses in the brain/CNS (*n* = 3), abdomen (*n* = 2), and lymph nodes (*n* = 1), alongside one case of combined local and bone relapse.

Among the 17 patients who experienced a local failure component (including one with combined local and bone relapse), all had received definitive radiotherapy alone without primary surgical resection as their initial local control modality. When stratified by site, the majority of local failures occurred in patients with parameningeal tumors (*n* = 9), followed by non-parameningeal/non-orbital (*n* = 5) and orbital primaries (*n* = 3).

**Figure 2:**
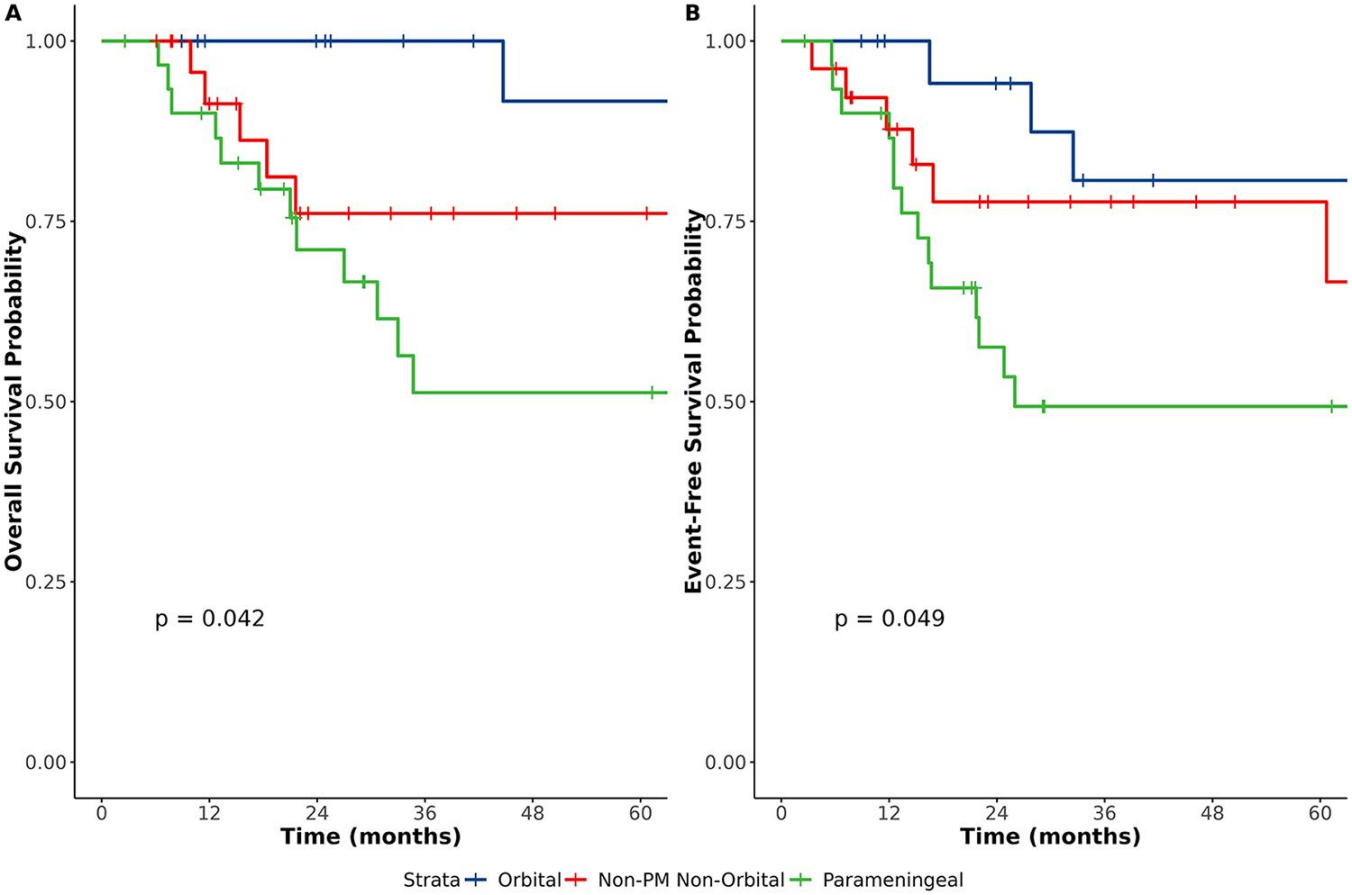
Kaplan–Meier 5-year event free survival and overall survival curves for patients with non-metastatic head and neck rhabdomyosarcoma according to primary sites.

Survival differed significantly according to primary tumor site. Orbital tumors demonstrated the most favorable outcomes with 5-year EFS and OS of 80.7 ± 10.1% and 91.7 ± 8%, respectively. Non-parameningeal/non-orbital tumors had 5-year EFS and OS of 73.7 ± 8.8% and 76.1 ± 9.4%, respectively, whereas parameningeal tumors showed inferior survival with 5-year EFS and OS 49.3 ± 9.7% and 51.2 ± 10.6% (EFS *p* = 0.049; OS *p* = 0.042) (Figure 2).

Univariable Cox regression analysis identified primary tumor site as a significant predictor of outcome. Patients with parameningeal tumors had significantly inferior OS compared with orbital tumors (HR: 5.21, 95% CI: 1.16–23.34, *p* = 0.03), and worse EFS (HR: 3.90, 95% CI: 1.12–13.63, *p* = 0.03). Larger tumor size (≥5 cm) showed a borderline association with inferior EFS (HR: 3.37, 95% CI: 1.00–11.38, *p* = 0.05) but not for OS (HR: 2.64, 95% CI: 0.77–9.08, *p* = 0.12).

In multivariable analysis, parameningeal location remained an independent adverse prognostic factor for OS in the extended model (HR: 8.77, 95% CI: 1.54–49.92, *p* = 0.01), whereas non-parameningeal non-orbital tumors did not show a statistically significant difference compared with orbital tumors. Only parameningeal location retained independent statistical significance for EFS in the multivariable model (Table 2).

**Table 2:** Univariate and multivariate cox model for event-free survival (EFS) and overall survival (OS) for all patients.

Parameter		EFS univariate		EFS multivariate		OS univariate		OS multivariate	
		HR, 95% CI	*p*-value	HR, 95% CI	*p*-value	HR, 95% CI	*p*-value	HR, 95% CI	*p*-value
Gender	Female vs. Male	0.91 (0.40–2.06)	0.82	—	—	0.90 (0.36–2.21)	0.81	—	—
Age at diagnosis	Mean (4.6 year)	0.93 (0.84–1.03)	0.17	0.94 (0.85–1.05)	0.29	0.93 (0.83–1.04)	0.22	0.93 (0.83–1.05)	0.23
Site	Orbital								
	Parameningeal	3.90 (1.12–13.6)	0.03	4.37 (1.00–19.11)	0.05	5.21 (1.16–23.34)	0.03	8.77 (1.54–49.92)	0.01
	Non-PM Non-Orbital	1.97 (0.49–7.89)	0.34	1.97 (0.49–7.89)	0.34	2.60 (0.50–13.43)	0.25	3.17 (0.55–18.09)	0.20
Tumor size	≥5 cm vs. <5 cm	3.37 (1.0–11.38)	0.05	3.71 (0.84–16.45)	0.08	2.64 (0.77–9.08)	0.12	3.63 (0.77–17.07)	0.1
Surgery	Yes vs. No	0.15 (0.02–1.13)	0.07	—	—	0.20 (0.03–1.48)	0.12	0.38 (0.07–2.00)	0.25
LN involvement	Positive vs. Negative	0.45 (0.11–1.93)	0.28	0.40 (0.08–1.95)	0.26	0.57 (0.13–2.45)	0.45	—	—
Radiotherapy	Yes vs. No	1.68 (0.23–12.5)	0.61	—	—	1.30 (0.17–9.72)	0.80	—	—

EFS, event free survival; OS, overall survival; HR, hazards ratio; CI, confidence interval; LN, lymph node; PM, parameningeal.

## Discussion

HNRMS represents a clinically distinct subgroup within pediatric RMS due to its complex regional anatomy, proximity to vital neurovascular structures, and the inherent challenges associated with achieving effective local control while preserving both function and cosmesis (5, 6).

In this series, 93.5% of patients presented with IRS Clinical Group III disease and 71% with tumors exceeding 5 cm—proportions that substantially exceed those reported in COG and EpSSG cooperative group trials, where IRS Clinical Group III disease represents 60%–65% of cases and large tumors 40%–55%. Despite this adverse disease profile, 5-year overall survival reached 70.1%, equivalent to benchmark figures from those same cooperative series. This survival was achieved in a MENA referral setting which might be defined by late presentation. The implication is direct: the survival gap historically attributed to resource-limited and late-presenting settings may reflect differences in disease burden at diagnosis rather than in the effectiveness of treatment delivery.

The distribution of primary tumor sites observed in this cohort is consistent with the established pattern of HNRMS, where parameningeal and orbital tumors are predominant. Parameningeal and orbital primaries accounted for 40% and 26% of cases, respectively. These results are in alignment with findings from large international datasets (7).

Embryonal RMS comprised 66% of our cohort and alveolar 23%, consistent with published HN series (8–10). Histological subtype distribution did not differ significantly across primary sites (*p* = 0.053), indicating that site-related outcome differences in multivariable analysis are not attributable to underlying subtype imbalance. The predominance of embryonal histology—associated with a more favorable prognosis—likely contributes in part to our cohort's comparable survival despite advanced presentation (9, 11).

Tumor size is widely recognized as a significant prognostic factor in pediatric RMS. Multiple studies have identified a tumor size greater than 5 cm as an independent predictor of adverse outcomes in HNRMS, mainly increased risks of progression, local failure, and mortality (12–14). The most striking feature of this cohort's presentation is tumor burden at diagnosis: 71% had tumors >5 cm, compared with 40%–55% in COG and EpSSG series. Several factors likely contribute to this pattern of advanced presentation. Combined with 93.5% IRS Clinical Group III, this suggests systematic diagnostic delay—most likely from low primary-care awareness of pediatric soft-tissue tumors and the non-specific early symptoms of HNRMS (unilateral proptosis, nasal obstruction, or facial swelling often attributed initially to inflammatory disease). Although symptom duration and referral intervals were not systematically captured, diagnostic delay represents a probable explanation for this advanced presentation. Larger tumors were associated with inferior EFS in univariable analysis (HR: 3.37, *p* = 0.05), though this did not persist after adjustment, suggesting primary site and treatment modality mediate much of the size effect. Reducing diagnostic delays through targeted education of primary care physicians and pediatricians should be a regional public health priority.

The management of HNRMS requires a carefully coordinated multidisciplinary approach (15). Given the anatomical complexity of the HN region, treatment planning must balance optimal oncologic control with the preservation of function and cosmesis (16). Current evidence consistently supports multimodal therapy, which integrates chemotherapy, radiotherapy, and selectively applied surgery. These comprehensive strategies have been associated with significant improvements in local control and survival over time (6, 15).

Radiotherapy (RT) is integral to local control in HNRMS, particularly at parameningeal and orbital sites where surgery is often not feasible (12). The significance of integrating systemic therapy with RT for local disease control has been recognized since the early cooperative group studies and remains a cornerstone of contemporary RMS management (17). Multiple studies have demonstrated that the inclusion of RT significantly improves local control and survival outcomes compared to approaches that omit radiation (12). Furthermore, modern RT techniques, such as intensity-modulated radiotherapy, have reported high rates of local control while minimizing toxicity in children with HNRMS (18–20). Despite these advancements, adverse factors such as intracranial extension and delays in RT initiation remain associated with an increased risk of local failure, underscoring the importance of timely and adequately administered RT for parameningeal disease (21).

The significance of RT is especially evident in parameningeal RMS, where surgical options are frequently limited. Early findings from the Intergroup Rhabdomyosarcoma Study highlighted the necessity of RT in achieving durable local control, with reported 5-year local control rates exceeding 80% (21). Subsequent prospective studies confirmed that the combination of intensive chemotherapy with appropriately timed RT could achieve local control rates exceeding 90% in selected patients (22). Our local failure rate of 22%—concentrated in parameningeal tumors (9 of 17 local failures)—is consistent with published rates of 10%–20% and underscores the persistent challenge of durable local control at this site.

A key observation is that OS equivalent to full-dose international protocols was maintained after reducing cyclophosphamide from 2.2 to 1.2 g/m^2^ in 2006, extending our previously reported institutional finding to the HN subsite over a 24-year follow-up (4). This has direct relevance for MENA and LMIC oncologists, where cumulative cyclophosphamide toxicity—particularly infertility and secondary malignancy—is a substantial management concern. From 2018, maintenance vinorelbine–cyclophosphamide was adopted for intermediate- and high-risk patients following the EpSSG RMS 2005 trial (23); 32% of the cohort received this regimen.

The 5-year OS of 70.1% and EFS of 66.1% are close to the COG D9803 series (5-year PFS ∼73%) and EpSSG RMS 2005 trial (5-year OS ∼71% for localized HN disease) (8, 24). The key point is that this parity was achieved despite 93.5% IRS Clinical Group III and 71% tumors >5 cm—substantially higher than cooperative group series—and in the absence of high-risk (metastatic) patients, meaning our composition should, if anything, have favored comparability.

Site-stratified outcomes are similar to published patterns: orbital 91.7% (reported 85%–92%) (25), non-parameningeal/non-orbital 76.1% (reported 70%–85%); parameningeal 51.2% (reported 34%–60%). The parameningeal deficit—driven entirely by local failure—is the finding that most requires intervention.

For orbital tumors, multiple series confirm comparable survival between surgery-based and radiotherapy-based local therapy (25–28); choice of modality is appropriately guided by functional and cosmetic considerations. Our 19% surgical rate and 92% radiotherapy rate reflect this: definitive RT spares orbital patients enucleation, surgery is avoided at parameningeal sites due to skull-base proximity, and resection at other sites is reserved for cases where gross total resection is achievable without major morbidity.

The higher proportion of alveolar histology observed among orbital tumors in our cohort compared with some international series may reflect sampling variation attributable to the relatively small number of orbital cases (*n* = 20). Population-based studies from North America and Europe report embryonal histology as predominant across all HN subsites including the orbit, with alveolar tumors constituting a minority. However, institutional series from the Middle East and Asia have occasionally reported a higher alveolar fraction, possibly reflecting differences in underlying biological heterogeneity or referral patterns rather than a true epidemiological difference (29, 30). This observation warrants validation in larger regional datasets and highlights the value of multicenter collaboration to characterize site-specific histological distributions in Middle Eastern pediatric populations.

Parameningeal RMS has historically been considered an unfavorable prognostic subgroup compared with other HN primary sites. However, accumulating evidence from cooperative group and institutional analyses indicates that the inferior outcomes traditionally attributed to parameningeal tumors are largely driven by differences in disease burden and adverse clinical features at presentation, rather than tumor location alone. Several studies have identified factors such as intracranial extension, skull base involvement, cranial nerve palsy, tumor size, and extent of local invasion as key determinants of prognosis. Patients without these high-risk features have demonstrated substantially improved survival, in some cases approaching outcomes observed in other HN primary sites (31, 32).

Local failure is the most common pattern of relapse in pediatric HNRMS, occurring more frequently than distant metastasis. Despite advances in radiotherapy, local progression remains a significant clinical challenge, particularly in patients with lymph node involvement at diagnosis (19, 33–35). Reported overall local failure rates in pediatric HNRMS range from approximately 10% to 20%. In our cohort, 17 of 23 patients (74%) with recurrence, representing 22% of the entire cohort, experienced local failure, a rate consistent with previous reports. These findings emphasize the ongoing need for improved strategies to achieve effective local control. Comparative data from the broader Middle East and South Asian region are emerging and provide important context for our findings. A recent multicenter analysis from the region documented patterns of RMS presentation and outcomes across multiple institutions, reporting similarly high rates of locally advanced disease and a predominance of parameningeal and orbital primaries, consistent with our cohort (30). An additional institutional series from Turkey focusing on orbital RMS further corroborated the generally favorable prognosis of orbital tumors, with 5-year OS rates comparable to our observed 91.7%, supporting the notion that orbital RMS outcomes are largely independent of geographic or resource setting when standard treatment protocols are applied (36). Together, these regional data suggest that the patterns observed at KHCC are not idiosyncratic but reflect broader epidemiological and clinical trends across this geographic area, and underscore the importance of building a cohesive regional evidence base.

The practical significance of this study lies not in confirming what cooperative group trials have established, but in demonstrating that their protocols are feasible and effective in a setting where non-metastatic patients present with substantially higher tumor burden—93.5% IRS Clinical Group III, 71% tumors >5 cm—than in typical cooperative group enrollments, and where molecular profiling is limited and cyclophosphamide dose is reduced. For MENA and LMIC oncologists, these data directly support COG-based multimodal treatment as the standard even in this high-burden non-metastatic context.

Our 5-year OS of 70.1% compares favorably with regional series reporting comparable non-metastatic cohorts: Markiz et al. reported 64.3% in Saudi Arabia (30, 37) and Badr et al. described broadly similar outcomes from Egypt (38). The higher OS at KHCC likely reflects consistent COG protocol adherence and an established MDT infrastructure. These regional comparisons are limited by differences in staging and metastatic exclusion criteria across series; a prospective MENA registry with standardized inclusion criteria would enable more rigorous comparison.

The multivariable Cox regression results should be interpreted cautiously because only 19 deaths and 23 events occurred in the study. With this small number of events, the hazard ratio estimates may be less reliable and can produce the wide confidence intervals observed (e.g., HR: 8.77, 95% CI: 1.54–49.92 for the parameningeal site). We therefore describe parameningeal location as the only factor reaching statistical significance in this cohort, rather than concluding that it is the sole independent prognostic factor. Larger studies are needed to confirm this finding.

This study does not generate new hypotheses about RMS biology; *n* = 77 cannot provide the statistical power of cooperative group trials. Its contribution is epidemiological and programmatic: documenting that a specific MENA population presents with substantially higher tumor burden than comparable non-metastatic Western series, yet achieves comparable survival with adapted protocols. The actionable implication—investment in early detection rather than intensification of systemic therapy—requires exactly this kind of real-world institutional data. Additional limitations include, first, the retrospective design with selection and ascertainment biases; second, 24-year accrual spanning three protocol generations and evolving supportive care; third, single-center origin limiting MENA generalizability; and fourth, incomplete FOXO1 fusion data for earlier-era patients. Based on the current WHO classification of soft tissue and bone tumors, FOXO1 and other molecular statuses are key prognostic discriminators. The absence of comprehensive molecular characterization in the majority of our patients represents a significant limitation of this study; and finally, the absence of systematic late-effects documentation.

## Conclusion

These findings confirm that COG-based multimodal treatment achieves outcomes equivalent to international benchmarks even when non-metastatic patients present with high tumor burden, and identify three priorities: intensification of local control for parameningeal disease; universal FOXO1 fusion testing to enable biology-driven risk stratification; and a prospective MENA registry including systematic capture of symptom-to-referral intervals to characterize disease-burden patterns at presentation.

## Data Availability

The raw data supporting the conclusions of this article will be made available by the authors, without undue reservation.

## References

[1] Crist W Gehan EA Ragab AH Dickman PS Donaldson SS Fryer C. The third intergroup rhabdomyosarcoma study. J Clin Oncol. (1995) 13:610–30. 10.1200/JCO.1995.13.3.610 7884423

[2] Chen E Ricciotti R Futran N Oda D. Head and neck rhabdomyosarcoma: clinical and pathologic characterization of seven cases. Head Neck Pathol. (2017) 11:321–6. 10.1007/s12105-016-0771-0 27896667 PMC5550390

[3] Sultan I Qaddoumi I Yaser S Rodriguez-Galindo C Ferrari A. Comparing adult and pediatric rhabdomyosarcoma in the surveillance, epidemiology and End results program, 1973 to 2005: an analysis of 2,600 patients. J Clin Oncol. (2009) 27:3391–7. 10.1200/JCO.2008.19.7483 19398574

[4] Al-Jumaily U Ayyad O Masarweh M Ghandour K Almousa A Al-Hussaini M. Improved care of rhabdomyosarcoma in Jordan using less intensive therapy. Pediatr Blood Cancer. (2013) 60:53–8. 10.1002/pbc.24241 22745011

[5] Radzikowska J Kukwa W Kukwa A Czarnecka AM Kawecki M Lian F. Management of pediatric head and neck rhabdomyosarcoma: a case-series of 36 patients. Oncol Lett. (2016) 12:3555–62. 10.3892/ol.2016.5072 27900036 PMC5104052

[6] Casey D Wolden S. Rhabdomyosarcoma of the head and neck: a multimodal approach. J Neurol Surg B. (2018) 79:058–64. 10.1055/s-0037-1617450 PMC5796820 29404242

[7] Owosho AA Huang S-C Chen S Kashikar S Estilo CL Wolden SL. A clinicopathologic study of head and neck rhabdomyosarcomas showing FOXO1 fusion-positive alveolar and MYOD1 -mutant sclerosing are associated with unfavorable outcome. Oral Oncol. (2016) 61:89–97. 10.1016/j.oraloncology.2016.08.017 27688110 PMC5097864

[8] Darwish C Shim T Sparks AD Chillakuru Y Strum D Benito DA. Pediatric head and neck rhabdomyosarcoma: an analysis of treatment and survival in the United States (1975–2016). Int J Pediatr Otorhinolaryngol. (2020) 139:110403. 10.1016/j.ijporl.2020.110403 33049553

[9] Reilly BK Kim A Peña MT Dong TA Rossi C Murnick JG. Rhabdomyosarcoma of the head and neck in children: review and update. Int J Pediatr Otorhinolaryngol. (2015) 79:1477–83. 10.1016/j.ijporl.2015.06.032 26231745

[10] Hicks J Flaitz C. Rhabdomyosarcoma of the head and neck in children⋆⋆presented at: american academy of oral and maxillofacial pathology, annual meeting essay section, Chicago, Illinois, may 2001. Oral Oncol. (2002) 38:450–9. 10.1016/S1368-8375(01)00105-1 12110339

[11] Perez EA Kassira N Cheung MC Koniaris LG Neville HL Sola JE. Rhabdomyosarcoma in children: a SEER population based study. J Surg Res. (2011) 170:e243–51. 10.1016/j.jss.2011.03.001 21529833

[12] Wen Y Huang D Zhang W Zhang Y Hu H Li J. Radiation therapy is an important factor to improve survival in pediatric patients with head and neck rhabdomyosarcoma by enhancing local control: a historical cohort study from a single center. BMC Pediatr. (2020) 20:265. 10.1186/s12887-020-02165-y 32471472 PMC7260775

[13] Ferrari A Miceli R Meazza C Zaffignani E Gronchi A Piva L. Soft tissue sarcomas of childhood and adolescence: the prognostic role of tumor size in relation to patient body size. J Clin Oncol. (2009) 27:371–6. 10.1200/JCO.2007.15.4542 19064986

[14] Raney RB Maurer HM Anderson JR Andrassy RJ Donaldson SS Qualman SJ. The intergroup rhabdomyosarcoma study group (IRSG): major lessons from the IRS-I through IRS-IV studies as background for the current IRS-V treatment protocols. Sarcoma. (2001) 5:9–15. 10.1080/13577140120048890 18521303 PMC2395450

[15] Meazza C Ferrari A Casanova M Massimino M Luksch R Spreafico F. Rhabdomyosarcoma of the head and neck region: experience at the pediatric unit of the istituto nazionale tumori, milan. J Otolaryngol. (2006) 35:53. 10.2310/7070.2005.4091 16527019

[16] Gillespie MB Marshall DT Day TA Mitchell AO White DR Barredo JC. Pediatric rhabdomyosarcoma of the head and neck. Curr Treat Options Oncol. (2006) 7:13–22. 10.1007/s11864-006-0028-3 16343365

[17] Tefft M Lindberg RD Gehan EA. Radiation therapy combined with systemic chemotherapy of rhabdomyosarcoma in children: local control in patients enrolled in the intergroup rhabdomyosarcoma study. Natl Cancer Inst Monogr. (1981) 56:75–81. 7029298

[18] Combs SE Behnisch W Kulozik AE Huber PE Debus J Schulz-Ertner D. Intensity modulated radiotherapy (IMRT) and fractionated stereotactic radiotherapy (FSRT) for children with head-and-neck-rhabdomyosarcoma. BMC Cancer. (2007) 7:177. 10.1186/1471-2407-7-177 17854490 PMC2077337

[19] Curtis AE Okcu MF Chintagumpala M Teh BS Paulino AC. Local control after intensity-modulated radiotherapy for head-and-neck rhabdomyosarcoma. Int J Radiat Oncol Biol Phys. (2009) 73:173–7. 10.1016/j.ijrobp.2008.03.029 18501529

[20] Frankart AJ Breneman JC Pater LE. Radiation therapy in the treatment of head and neck rhabdomyosarcoma. Cancers (Basel). (2021) 13:3567. 10.3390/cancers13143567 34298780 PMC8305800

[21] Michalski JM Meza J Breneman JC Wolden SL Laurie F Jodoin MA. Influence of radiation therapy parameters on outcome in children treated with radiation therapy for localized parameningeal rhabdomyosarcoma in intergroup rhabdomyosarcoma study group trials II through IV. Int J Radiat Oncol Biol Phys. (2004) 59:1027–38. 10.1016/j.ijrobp.2004.02.064 15234036

[22] Douglas JG Arndt CAS Hawkins DS. Delayed radiotherapy following dose intensive chemotherapy for parameningeal rhabdomyosarcoma (PM-RMS) of childhood. Eur J Cancer. (2007) 43:1045–50. 10.1016/j.ejca.2007.01.033 17368885

[23] Bisogno G De Salvo GL Bergeron C Gallego Melcón S Merks JH Kelsey A. Vinorelbine and continuous low-dose cyclophosphamide as maintenance chemotherapy in patients with high-risk rhabdomyosarcoma (RMS 2005): a multicentre, open-label, randomised, phase 3 trial. Lancet Oncol. (2019) 20:1566–75. 10.1016/S1470-2045(19)30617-5 31562043

[24] Curry SD Jiang ZY Jain KS. Population-Based survival of pediatric rhabdomyosarcoma of the head and neck over four decades. Int J Pediatr Otorhinolaryngol. (2021) 142:110599. 10.1016/j.ijporl.2020.110599 33422992

[25] Tang L-Y Zhang M-X Lu D-H Chen Y-X Liu Z-G Wu S-G. The prognosis and effects of local treatment strategies for orbital embryonal rhabdomyosarcoma: a population-based study. Cancer Manag Res. (2018) 10:1727–34. 10.2147/CMAR.S163932 29983592 PMC6025768

[26] Oberoi S Lambert P Gupta AA Deyell RJ Sung L Cuvelier GDE. Diagnostic and treatment intervals are not associated with survival in rhabdomyosarcoma: a cancer in young people in Canada study. Pediatr Blood Cancer. (2022) 69:e29306. 10.1002/pbc.29306 34455698

[27] Schoot RA Saeed P Freling NJ Blank LECM Pieters BR Van Der Grient JNB. Local resection and brachytherapy for primary orbital rhabdomyosarcoma: outcome and failure pattern analysis. Ophthal Plast Reconstr Surg. (2016) 32:354–60. 10.1097/IOP.0000000000000562 26398242

[28] Al-Bdour M Odat RM Salaimeh N Hamdah LA Ibrahim J Zidan A. 77P Clinical characteristics and effects of treatment strategies for patients with orbital rhabdomyosarcoma: a retrospective study. ESMO Open. (2024) 9:102467. 10.1016/j.esmoop.2024.102467

[29] Ramanathan S Sisodiya S Shetty O Prasad M Parambil BC Shah S. Outcome and prognostic variables in childhood rhabdomyosarcoma (RMS) with emphasis on impact of FOXO1 fusions in non-metastatic RMS: experience from a tertiary cancer centre in India. ecancermedicalscience. (2023) 17:1539. 10.3332/ecancer.2023.1539 37138963 PMC10151086

[30] Markiz SN Khan S Wagley ZB Viqaruddin MK Khafaga YM AlFawaz IA. Rhabdomyosarcoma in children: retrospective analysis from a single tertiary care center in Saudi Arabia. Cancer Rep. (2023) 6:e1683. 10.1002/cnr2.1683 PMC9875671 35942988

[31] Merks JHM De Salvo GL Bergeron C Bisogno G De Paoli A Ferrari A. Parameningeal rhabdomyosarcoma in pediatric age: results of a pooled analysis from North American and European cooperative groups. Ann Oncol. (2014) 25:231–6. 10.1093/annonc/mdt426 24356633 PMC3868324

[32] Hawkins DS Anderson JR Paidas CN Wharam MD Qualman SJ Pappo AS. for the Intergroup Rhabdomyosarcoma Study Group of the Children’s Oncology Group. Improved outcome for patients with middle ear rhabdomyosarcoma: a children’s oncology group study. J Clin Oncol. (2001) 19:3073–9. 10.1200/JCO.2001.19.12.3073 11408504

[33] Wharam MD Meza J Anderson J Breneman JC Donaldson SS Fitzgerald TJ. Failure pattern and factors predictive of local failure in rhabdomyosarcoma: a report of group III patients on the third intergroup rhabdomyosarcoma study. J Clin Oncol. (2004) 22:1902–8. 10.1200/JCO.2004.08.124 15143083

[34] Ludmir EB Grosshans DR McAleer MF McGovern SL Harrison DJ Okcu MF. Patterns of failure following proton beam therapy for head and neck rhabdomyosarcoma. Radiother Oncol. (2019) 134:143–50. 10.1016/j.radonc.2019.02.002 31005208

[35] Vern-Gross TZ Indelicato DJ Bradley JA Rotondo RL. Patterns of failure in pediatric rhabdomyosarcoma after proton therapy. Int J Radiat Oncol Biol Phys. (2016) 96:1070–7. 10.1016/j.ijrobp.2016.08.028 27742542

[36] Ulas B Ozcan AA Aljundi S. Orbital rhabdomyosarcoma: clinicodemographic features and outcomes from Turkey. Indian J Ophthalmol. (2025) 73:1132–7. 10.4103/IJO.IJO_921_24 40719714 PMC12416614

[37] Totadri S Bansal D Donaldson SS Binitie O Teot L Gupta AA. Common queries in managing rhabdomyosarcoma in low- and middle-income countries: an indo-North American collaboration. Pediatr Blood Cancer. (2023) 70:e30616. 10.1002/pbc.30616 37574816

[38] Badr MA Al-Tonbary YA Mansour AK Hassan TH Beshir MR Darwish A. Epidemiological characteristics and survival studies of rhabdomyosarcoma in east Egypt: a five-year multicenter study. ISRN Oncol. (2012) 2012:1–8. 10.5402/2012/674523 PMC3362855 22675642

